# BDNF, Brain, and Regeneration: Insights from Zebrafish

**DOI:** 10.3390/ijms19103155

**Published:** 2018-10-13

**Authors:** Carla Lucini, Livia D’Angelo, Pietro Cacialli, Antonio Palladino, Paolo de Girolamo

**Affiliations:** 1 Department of Veterinary Medicine, University of Naples Federico II, 80137 Naples, Italy; livia.dangelo@unina.it (L.D.); Pietro.Cacialli@unige.ch (P.C.); paolo.degirolamo@unina.it (P.d.G.); 2 Stazione Zoologica Anton Dohrn, 80122 Naples, Italy; 3 Centro Ricerche Interdipartimentali sui Biomateriali, University of Naples FedericoII, 80125 Naples, Italy; a.palladino1986@gmail.com

**Keywords:** Fish, neuroregeneration, neurotrophins, Trk B, p75

## Abstract

Zebrafish (*Danio rerio*) is a teleost fish widely accepted as a model organism for neuroscientific studies. The adults show common basic vertebrate brain structures, together with similar key neuroanatomical and neurochemical pathways of relevance to human diseases. However, the brain of adult zebrafish possesses, differently from mammals, intense neurogenic activity, which can be correlated with high regenerative properties. Brain derived neurotrophic factor (BDNF), a member of the neurotrophin family, has multiple roles in the brain, due also to the existence of several biologically active isoforms, that interact with different types of receptors. BDNF is well conserved in the vertebrate evolution, with the primary amino acid sequences of zebrafish and human BDNF being 91% identical. Here, we review the available literature regarding BDNF in the vertebrate brain and the potential involvement of BDNF in telencephalic regeneration after injury, with particular emphasis to the zebrafish. Finally, we highlight the potential of the zebrafish brain as a valuable model to add new insights on future BDNF studies.

## 1. The Brain of the Zebrafish Is a Model for Neuroscientific Studies

Zebrafish (*Danio rerio*) is a teleost fish and has become an interesting model organism in biomedical research due to its genetic tractability, small size, and easy maintenance and breeding. The zebrafish genome, fully sequenced, is highly homologous to humans, since 70% of human genes (and 82% of disease-causing human proteins) have at least one zebrafish orthologue [1]. Overall physiology of the zebrafish resembles human physiology, allowing the use of this model for disease modeling, such as metabolic [2], hematopoetic [3], and cardiovascular [4] disorders. The neuroanatomy of the zebrafish brain has been extensively described and atlases of the brain are available for adults [5] and embryos [6]. The brain structure resembles that of mammals [7]. The same neurotransmitters in both interneuron systems and in long pathways have been described [8], and several areas show homologous functions [9,10]. For these reasons, in the last decade, the zebrafish has been accepted as a model to study the molecular and cellular biology of the vertebrate brain [11,12], and emerged for translational research in various neurological and neuropsychiatric conditions [13,14], such as epilepsy [15,16], Alzheimer’s [17] and Parkinson’s diseases [18], depressive disorders [19], schizophrenia [20], and autism spectrum disorders [21]. Also, the zebrafish is a robust model for studying the effects of metabolic disorders on the central nervous system, as acute and chronic hyperglycemia appear to impact on brain homeostasis and neurogenesis [22].

## 2. The Evolution of the Brain Derived Neurotrophic Factor (BDNF) Gene and Protein

BDNF belongs to the neurotrophin family, whose other members are nerve growth factor (NGF), neurotrophin (NT) 3, and NT 4/5. Nowadays, BDNF has retained an ancient presence in animal evolutive lineage. Vertebrate-like representatives of neurotrophin signaling molecules have been hypothesized in invertebrates [23,24,25,26,27,28,29,30,31] and in the cephalochordate amphiouxus [32]. Presumably, from two intermediate neurotrophin gene ancestors, the couple, NGF and NT3, as well as BDNF and NT4/5, were formed after the split of jawless fish, but before the split of cartilaginous fish from the common vertebrate lineage [33,34]. In bony fish, an additional duplication was suggested: The same ancestor gave origins by duplication to NGF and NT 6/7, the latter a neurotrophin not known in other vertebrates [35,36,37].

Phylogenetically, BDNF is well conserved. The zebrafish BDNF gene has a more compact organization than that of mammalians. Moreover, the promoter structure and patterns of differential promoters’ expression are as complex as in the murine counterpart [38].

In mammals, three different forms derive from the *bdnf* gene: The preproBDNF, proBDNF, and mature BDNF [39]. The preproBDNF carries a signal peptide that is cleaved off, forming proBDNF. The latter is further processed to generate the mature BDNF. Both pro and mature BDNF are secreted. Also, prodomain recently resulted in a detectable protein and undergoes activity-dependent secretion from hippocampal neurons [40]. PreproBDNF for each class of vertebrates has been described and their alignment against the preproprotein of *Homo sapiens* demonstrated high conservative sequences. In zebrafish, three isoforms of proBDNF have been reported (NP_001295577.1, NP_001295578.1, NP_571670.2), with around 95% of identity [41]. Finally, the DNA-deduced amino acid sequence of the processed mature BDNF in the teleost fish, *Xiphophorus maculatum*, showed 90% identity with the mouse sequence [42], and the primary amino acid sequences of zebrafish and human BDNF are 91% identical [43,44].

## 3. Different Receptors Mediate the Multiple Roles of BDNF in the Brain

Several biologically active BDNF isoforms (prepro- pro-, mature-BDNF) interact with different types of receptors. Mature BDNF can bind to two TrkB receptor isoforms abundantly expressed in the brain: Full-length TrkB and truncated TrkB. The full length TrkB, activated by mature BDNF, can transduce intracytoplasmatically the signal. The truncated TrkB lacks the intracellular kinase domain and thus cannot undergo autophosphorylation, but it binds to and internalizes BDNF, acting as a dominant-negative receptor that indirectly inhibits BDNF function. However, recent studies indicate that truncated TrkB sequesters and translocates BDNF, induces neurite outgrowth, and regulates cytoskeletal changes in astrocytes and glioma cells [45]. Also, mature BDNF binding to dimerized p75NTR may enhance neurotrophin binding to Trk receptors, and further promote survival. Pro-BDNF binding to p75NTR associated to sortilin induces apoptosis [46]. p75NTR can also form part of the Nogo receptor complex, activated by myelin proteins, to inhibit axonal growth [47]. In teleost fish, two types of receptors are known. The TrkB receptor, due to a specific genome duplication [48], is present as TrkB1 and TrkB2 [49]. The amino acid residues of the kinase domain involved in signal transduction are identical in zebrafish and mammals [44]. Full-length zebrafish p75 cDNA was cloned and is predicted to encode a protein with 61% similarity to both human and mouse p75. Four cysteine-rich domains (CRD1-4) were identified in the extracellular region, and a death domain at the C-terminal tail of the intracellular region of zebrafish p75 [50].

As result, multiple BDNF isoforms, transduced by different types of receptors, exert multiple and important roles. During mammalian brain development, BDNF regulates neuro-, glio-, and synaptogenesis, and the elimination of improperly formed connections [51]. Attenuation of BDNF signaling as a consequence of prolonged maternal separation profoundly affects hippocampal circuitry [52]. In adulthood, prevailing BDNF processes enhance the efficiency of stimulus transmission and synaptic plasticity, which support memory and cognition [52,53]. Indirect evidence is given by a common single-nucleotide polymorphism in the human *bdnf* gene, resulting in a valine to methionine substitution in the pro-domain (Val66Met), which leads to memory impairment and susceptibility to neuropsychiatric disorders [54,55]. In patients with schizophrenia, BDNF mRNA serum levels were reduced [56]. Depressive disorders and other mood diseases could be underlined by the disturbance of tight and well-balanced associations between BDNF and the excitatory neurotransmitter glutamate systems [57]. In depressed patients, BDNF levels appeared lower, and treatment with antidepressants increased serum BDNF levels compared to controls [58]. Also, recent findings indicate a role of BDNF in the pathophysiology of autism spectrum disorder [59]. BDNF also has beneficial effects. In Alzheimer disease, in vitro and in vivo studies in rats indicated that BDNF has neuronal protective effects against neurotoxicity caused by amyloid β-peptide accumulation. In fact, BDNF could act as an antioxidative factor since it is known that it increases the level of activity of some antioxidant enzymes [60]. Finally, in the treatment of multiple sclerosis, BDNF plays a role in the mechanism of re-myelination [61].

## 4. The Distribution of BDNF in the Brain of Vertebrates

The distribution of BDNF mRNA and protein has been described in the brain of different mammalian and non-mammalian species during development and adulthood.

In rats, *bdnf* expression dramatically increased between embryonic days 11 and 12, a timing coinciding with maturation of cerebral regions, and gained high levels in hippocampus [62]. In the adult mouse, *bdnf* is expressed mostly in the hippocampus, followed by the cerebral cortex [63]. In adult rats, BDNF mRNA and protein are expressed in the hippocampus, septum, cerebral cortex, adrenergic nuclei of the brainstem, and in the hypothalamus [64,65]. Particularly, BDNF protein appears to be more widely diffused than BDNF mRNA [65]. Furthermore, Conner and collaborators [66], by comparing the distribution of BDNF mRNA and protein, suggested an anterograde transport of BDNF protein in some afferent systems. In the human brain, BDNF messengers were also reported in the hippocampus, amygdala, and septum [67,68]. In songbirds, *bdnf* is expressed in nuclei involved in sensorimotor integration of song learning [69,70,71]. In pigeons, BDNF protein is involved in the retino-tectal system of adults [72]. In amphibians, BDNF mRNA and protein were described in the retino-tectal system of *Rana pipiens* [73]; and BDNF protein in the hypothalamo-hypophyseal system of *Xenopus laevis* [74].

In the fish brain, proBDNF was detected in the optic tect and hypothalamus of perciform *Cichlasoma dimerus* [75]. In the eel (*Anguilla anguilla*) [76,77], and Turquoise killifish (*Nothobranchius furzeri*) [78], BDNF mRNA was reported in the forebrain (olfactory bulb, dorsal and ventral telencephalon, preoptic area, epi-, hypo- thalamus, pretectum), midbrain (optic tect, tegmentum), hindbrain (cerebellum, reticular formation, octavolateral area), and spinal cord. Furthermore, in young and adult teleosts, BDNF was described in sensory organs in the inner ear [79], the lateral line system [80], retina [75,81,82,83], and gonads [84].

## 5. The Distribution of BDNF in the Brain of Zebrafish

In zebrafish, BDNF mRNA was detected early during embryonic development. Whole-mount in situ hybridization experiments demonstrated *bdnf* transcripts in the forebrain, midbrain, and hindbrain [43,85,86]. In the brain of seven day old larvae, BDNF mRNA was detected in the olfactory rosettes, dorsal telencephalon, preoptic and thalamic area, optic tect, tegmentum, and reticular formation [87].

**Figure 1:**
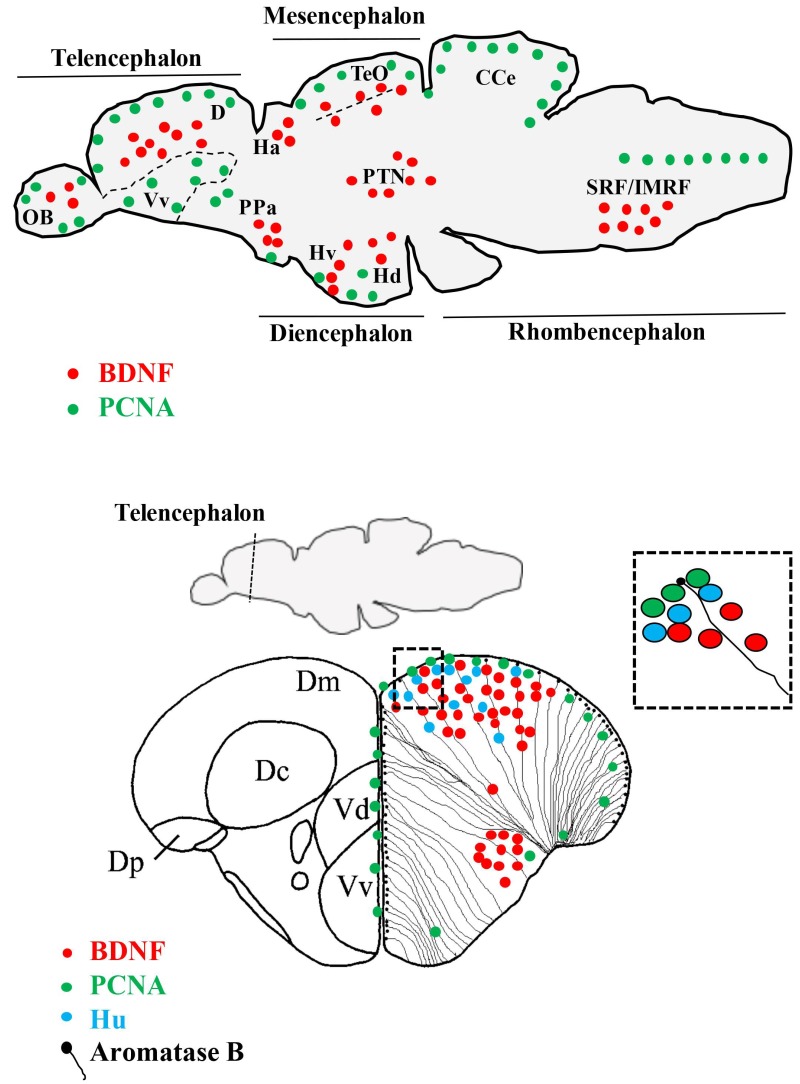
Brain derived neurotrophic factor (BDNF) mRNA distribution in sagittal and cross sections of the zebrafish brain. Cells containing BDNF mRNA (red dots) are mature neurons. In several areas, they are close to radial glial (black dots) and proliferating (green dots) cells and Hu positive (azure dots) young neurons. Abbreviations: CCe: Cerebellar body; D: Dorsal telencephalon; Dc: Central area of dorsal telencephalon; Dm: Medial area dorsal telencephalon; Dp: Posterior area of dorsal telencephalon; Ha: Abenula; Hd: Dorsal zone of periventricular hypothalamus; Hv: Ventral zone of periventricular hypothalamus; OB: Olfactory bulb; PPa: Anterior part of parvocellular preoptic nucleus; PTN: Posterior tuberal nucleus; SRF/IMRF: Superior/intermediate reticular formation; TeO: Optic tect; Vd: Dorsal nucleus of ventral telencephalic area; Vv: Ventral nucleus of ventral telencephalic area.

In the adult zebrafish brain, the localization of *bdnf* transcripts is distributed in all regions of the brain (Figure 1) [87], without significant differences between sex, although sex hormones have been recognized to influence the expression and activity of BDNF through a combination of genomic and epigenetic mechanisms. However, the impacts of estrogens on *bdnf* expression are complex and vary according to the species, age, brain region, and treatment [88,89,90].

BDNF mRNA is expressed in the neuronal population of an adult zebrafish brain, as coexistence of *bdnf* transcripts and the neuronal markers, HuC/D, MAP2, and acetylated-tubulin, demonstrated. Because the neuronal marker, HuC, specifically characterize early differentiated neurons [91], whereas the markers, MAP2 and acetylated-tubulin, characterize quite differentiated neurons [92,93], it can be retained that *bdnf* is expressed just after proliferative events, when positive cells start to assume neuronal phenotypes, and maintain the expression in mature neurons. Consistently, a recent study in zebra finch showed the presence of BDNF protein in newly generated cells marked with bromodeoxyuridine [70]. In some regions of the zebrafish brain, *bdnf* expressing cells were located close to the ventricular surface, where radial glial cells are usually distributed. These cells can be in a quiescent state or, being proliferative, give origins to new neurons [94]. The use of the glial marker, aromatase B, and brain lipid-binding protein (BLBP) suggested that radial glial cells do not express *bdnf* under physiological conditions. Furthermore, any co-expression of *bdnf* and the proliferation marker, PCNA, in the same cells was observed, corroborating the hypothesis that proliferating radial glial cells do not express BDNF. All together, these findings further confirm the neuronal nature of *bdnf* expressing cells, consistent with the fact that BDNF is of neuronal origins in mammals [64,68]. Nonetheless, under pathological conditions, such as after brain lesions or around amyloid plaques, *bdnf* expression has been documented in glial cells [69,95,96].

Considering in detail the distribution of BDNF mRNA in the adult zebrafish brain, *bdnf* expression is mostly seen in the forebrain and only in a few areas of the midbrain and hindbrain: The most prominent staining was seen in the dorsal telencephalon, epithalamus, posterior tuberculum, hypothalamus, and synencephalon. A more diffuse, but weaker, labelling is detected in other brain regions, such as the olfactory bulbs, ventral telencephalic area, preoptic area, dorsal- and ventral thalamus, optic tect, semicircular tori, tegmentum, cerebellum, and medulla oblongata [87]. In addition, in a morphological survey devoted to the presence of neurotrophins and their Trks receptors in the cerebellum, BDNF protein was seen in Purkinje cells of the valvula and body, as well as in fibers running in the granular cell layer [97].

Overall, BDNF distribution in zebrafish is similar to that of other teleosts [75,77,78], although differences should also be highlighted: (a) *bdnf* is expressed more in the ventral telencephalon of the European eel and the turquoise killifish; (b) the staining in the diencephalon appears more widespread in the zebrafish than in the eel; (c) intense positive labeling was seen in other diencephalic nuclei of the killifish, such as the nucleus cortical and nucleus glomerulus, which are lacking in the brain of the zebrafish; and (d) *bdnf*-expressing cells in the tegmentum and rhombencephalon were fewer in the zebrafish than in the eel and turquoise killifish.

Also, when comparing the distribution of *bdnf* messengers in the brain of zebrafish with that reported in mammals, the findings are quite similar [63,64,65,66]. Indeed, strong *bdnf* expression was reported in the cortex and hippocampus, two pallial structures. Because the telencephalon of fish develops by eversion, the hippocampus equivalent of zebrafish is the dorsolateral region of the dorsal telencephalon [98,99], where abundant *bdnf* messengers are detected. It is also noteworthy that the central part of the telencephalon, regarded as the presumptive equivalent of the isocortex of mammals, also strongly expresses *bdnf* [64]. Similarly to their mammalian counterparts, the preoptic area, and in particular the magnocellular neurons, of zebrafish express *bdnf* messengers. Other structures exhibiting *bdnf* mRNAs in both fish and mammals include the habenula, thalamic region, mediobasal hypothalamus, and optic tect (inferior colliculus). These similarities suggest that BDNF functions are conserved between zebrafish and mammals [87].

## 6. BDNF and Adult Neurogenesis, Neuroregeneration in Vertebrate Brain

The number of newly produced neurons and neurogenic niches in the adult brain decreases during evolution and, consequently, regenerative potentiality also dramatically declines [100].

In mammals, adult neurogenesis is quite limited and only two neurogenic regions are well described: The subventricular zone of the lateral ventricle and the subgranular zone of the dentate gyrus in the hippocampus. Moreover, newly formed neurons do not survive for a long time, likely due to a non-suitable local environment. Very recently, two studies reported contrasting results in humans: Sorells and coworkers [101] concluded that neurogenesis drops to undetectable amounts during childhood, whereas Boldrini and colleagues [102] reported lifelong neurogenesis [103].

Instead, the brain of adult zebrafish exhibits a high number of proliferative areas (Figure 1), mainly localized in 16 neurogenic niches [104]. The majority are distributed along the ventricles of the telencephalon, diencephalon, and mesencephalon [94,104,105,106,107], from which newly differentiated neurons start to migrate. Neurogenesis continues along the entire adult life, a feature tightly linked to the persistence of radial glial progenitors [108], which gives an outstanding capacity to regenerate after brain injury [24,109,110].

The impact of exogenous BDNF on neurogenesis was studied by infusion into the lateral ventricles of the adult rat. BDNF substantially increased the number of newborn cells in many regions, the preponderance of which differentiate into neurons [111,112]. BDNF administration in the hippocampus was associated with an increased neurogenesis of granule cells in the dentate gyrus [113]. However, successively, Galvao and colleagues [114] obtained contrasting results, suggesting that BDNF, delivered intracerebroventricularly in mice and rats, failed to enhance neurogenesis in the subventricular zone, but even reduced it. These contradictory results could be due to differences in the reagents being used by the various labs. It is known that the production of BDNF in some recombinant systems leads to misfolding of the protein and possibly changes in its efficacy to stimulate target receptors. In addition, differences in the abundance of the preprocessed pro form of BDNF relative to the mature form may also contribute to the differences in observed results [115].

The results regarding the action of endogenous BDNF on neurogenesis are quite complex and contrasting. Studies in heterozygous BDNF knockout mice reported that proliferation of neural stem cells was decreased in the dentate gyrus of the hippocampus [116]. In contrast, conditional knockout mice with a depletion of BDNF in mature neurons exhibited an increase in hippocampal proliferation [117].

Regenerative processes after damage, extremely scarce in the mammalian brain, seem to involve BDNF. In neonatal rats with left common carotid artery ligation to mimic hypoxic–ischemic (HI) encephalopathy, the levels of BDNF increased significantly after an HI event [118]. Traumatic brain injury (TBI) and post-traumatic stress disorders in humans have been related to dysregulation of BDNF [119]. Experimental severe TBI caused an increase of BDNF mRNAs and protein levels in rat brain homogenates [118]. In contrast, the mild severity level of TBI obtained by lateral percussion displayed unchanged BDNF mRNAs levels in hippocampal homogenates [120].

In rodents, the increase of BDNF mRNA expressing cells was usually reported in the contralateral side, at various time intervals from the injury [120,121,122,123]. However, within 24 h of the lesion, contrasting results were reported in these species: (a) Increase of BDNF mRNA expressing cells in the injury side [124,125]; (b) decrease of BDNF mRNA expressing cells in the injury side [120]; or (c) bilateral increase of BDNF mRNA expressing cells [126].

Regarding BDNF receptors, after the acute phase of experimental injury, the mRNA expression of full length and truncated TrkB and p75 receptors resulted increased [120]. As further evidence, p75 mutant mice as well as mice treated with the p75 antagonist or the TrkB agonist exhibited reduced post traumatic events, such as neuronal death and degeneration, and reduced astrocytosis [127,128]. In contrast, adult gerbil CA1 neurons, which showed BDNF and TrkB colocalization, remained resistant to damage during forebrain ischemia [129].

After TBI, BDNF appears to mediate some beneficial treatments, such as: (a) The transplantation of neural stem cell increased neurological function improvement, which was associated with the upregulation of synaptophysin and BDNF expression [130]; (b) transcranial ultrasound stimulation and transcranial low-level laser light therapy to the brain, partly mediated by stimulation of BDNF, seems to encourage synaptogenesis and to reduce apoptosis [131,132]; and (c) exercise, which upregulates BDNF within the hippocampus, is associated with an enhancement of cognitive recovery both in human and rodents [133]. However, the therapeutic potential of BDNF for TBI is restricted due to the short half-life and inability to cross the blood-brain barrier. Recently, however, the flavonoid, 7,8-dihydroxyflavone (7,8-DHF), a small TrkB agonist that mimics BDNF function, has shown effects in promoting neuronal survival and regeneration following TBI [134].

**Figure 2:**
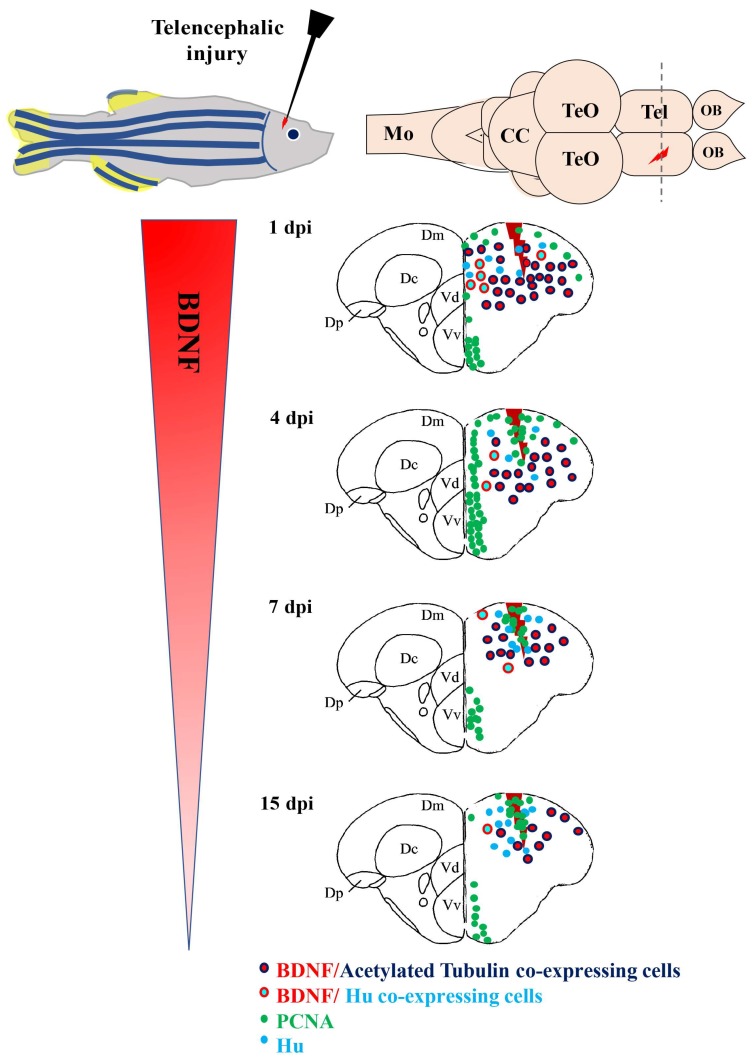
BDNF mRNA distribution in cross sections of the zebrafish telencephalon after injury. Cells containing BDNF mRNA (red dots) are young (Hu positive, azure dots) and mature (Acetylated-Tubulin, blue dots) neurons. In several areas, they resulted close to proliferating cells (green dots). Abbreviations: CC: Cerebellar body; Dc: Central area of dorsal telencephalon; Dm: Medial area of dorsal telencephalon; Dp: Posterior area of dorsal telencephalon; Mo: Medulla oblongata; OB: Olfactory bulb; Tel: Telencephalon; TeO: Optic tect: Vd: Dorsal nucleus of ventral telencephalic area; Vv: Ventral nucleus of ventral telencephalic area.

In contrast to mammals, profound stabs, which mimics severe TBI, on the zebrafish encephalon, revealed a series of regenerative processes. At the end of them, complete recovery of the lesioned area and mature neurons with marker profiles similar to preexisting neurons were reported [135]. Thus, the comparison of data obtained in zebrafish and mammals should reveal similar, as well as divergent, properties of adult neuroregeneration. BDNF involvement in the regenerative process of the zebrafish brain after a stab wound was recently reported [136,137]. The lesion was performed in the dorso-lateral telencephalon because this region comprises the most studied neuronal stem cell niches [104,135] and its dorso-lateral zone is retained to be equivalent to the medial pallium (hippocampus) of mammals [138], which contains one of the two constitutive neurogenic niches of mammals: The subgranular zone of the dentate gyrus. Immediately after the lesion, one day post lesion (dpl), a remarkable increase of BDNF mRNA levels in homogenates of the whole lesioned telencephalon occurred, and then BDNF mRNA levels decreased with time after the lesion. Consistently, BDNF mRNAs expressing cells in the whole dorsal telencephalon clearly increased in number at 1 dpl. These BDNF positive cells, in accordance with results regarding BDNF in un-lesioned animals, can be retained as mature neurons, as they contained acetylated-tubulin (a marker of mature neurons), rarely had HuC/D (a marker of early differentiated neurons), and never had PCNA (a proliferative marker) or co-expressed aromatase B. Thus, the increase of BDNF-expressing cells following injury resulted from mature neurons, which triggered BDNF translation after lesion, and not from newly generated neurons [136] (Figure 2).

In fact, because the maximum increase of BDNF mRNA expressing neurons occurred soon after the lesion, it is unlikely that neuronal precursor cells migrate from ventricular zone to the injury site, where they differentiate into mature neurons [135]. Finally, in zebrafish, the number of BDNF mRNA expressing cells decreased as time after the lesion passed, but remained significantly higher than in the control side. Thus, BDNF mRNAs expressing neurons were more numerous in the injured side compared to the contralateral [136]. Contrastingly, in rodents, BDNF expression persists only in the contralateral side because of neuronal loss and glial scar formation in the injured area. Considering the complete repair of the damaged area in zebrafish, BDNF could be considered as a factor contributing to create a permissive environment that enables the establishment of a new neuronal network in a damaged brain.

In fish, pro and mature BDNF proteins exist [38,41,42,43,44,139], and in homogenates’ brains of Turquoise killifish, both forms are present [78]. In adult zebrafish, the presence of the TrkB protein was generally reported in the brain [140] and in the cerebellum [97], and treatment with the selective TrkB antagonist, ANA-12, after stab injury reduced the proliferation activity in the adult zebrafish brain [141]. In addition, in the brain of the adult Turquoise killifish, the TrkB protein was identified in neurons of different regions of the forebrain and in radial glial cells lining the mesencephalic and rhombencephalic ventricles [142].

Finally, the increase of BDNF mRNA serum levels and expressing neurons after a lesion could be related to the inflammation process that occurs after telencephalic injury. In zebrafish, it has been observed that inflammation is required and induces the proliferation of neural progenitors and subsequent neurogenesis by activating injury-induced molecular programs that can be observed after 24–48 h post-traumatic brain injury [143]. Also, gene-regulatory network analysis revealed that the BDNF-TrkB signaling pathway positively regulated brain inflammation in zebrafish during seizures induced by pentylenetetrazole (PTZ). The use of K252a, a TrkB inhibitor, to block the BDNF-TrkB signaling pathway attenuated PTZ-induced seizures, confirming BDNF-TrkB mediated inflammatory responses [144].

## 7. Concluding Remarks

Evidence gathered in this review suggest that the zebrafish brain shows a high conservation of some brain structures, compared to mammals, together with similar key neuroanatomical and neurochemical pathways of relevance to human diseases. Differently from mammals, the zebrafish brain possesses intense neurogenesis that can be correlated with high regenerative properties. Recently, the zebrafish has been proposed as a valid experimental paradigm to study the association of brain derived neurotrophic factor (BDNF) and neural repair after traumatic brain injury. In this sense, it represents a valuable and alternative model to mammals.
